# The follicular lymphoma epigenome regulates its microenvironment

**DOI:** 10.1186/s13046-021-02234-9

**Published:** 2022-01-12

**Authors:** Rada Amin, Mounia S. Braza

**Affiliations:** 1 Department of Biochemistry, University of Nebraska-Lincoln, Lincoln, NE USA; 2 Department of Oncological Sciences, Icahn School of Medicine at Mount Sinai, New York, NY USA

**Keywords:** Hemato-oncology, Lymphoma, Immunotherapy, Epigenetic

## Abstract

Follicular lymphoma (FL) is a B-cell non-Hodgkin lymphoma of germinal center (GC) origin with a distinctive tumor microenvironment (TME) and a unique spectrum of mutations. Despite the important therapeutic advances, FL is still incurable. During B-cell development, the GC reaction is a complex multistep process in which epigenetic regulators dynamically induce or suppress transcriptional programs. In FL, epigenetic gene mutations perturb the regulation of these programs, changing GC B-cell function and skewing differentiation towards tumor cells and altering the microenvironment interactions. FL pathogenesis and malignant transformation are promoted by epigenetic reprogramming of GC B cells that alters the immunological synapse and niche. Despite the extensive characterization of FL epigenetic signature and TME, the functional consequences of epigenetic dysregulation on TME and niche plasticity need to be better characterized. In this review, first we describe the most frequent epigenomic alterations in FL (*KMT2D*, *CREBBP* and *EZH2*) that affect the immunological niche, and their potential consequences on the informational transfer between tumor B cells and their microenvironment. Then, we discuss the latest progress to harness epigenetic targets for inhibiting the FL microenvironment. Finally, we highlight unexplored research areas and outstanding questions that should be considered for a successful long-term treatment of FL.

## Background

Follicular lymphoma (FL) is the most frequent indolent B-cell non-Hodgkin lymphoma (yearly incidence: 2.7 cases per 1000,000 inhabitants in the USA in the 2014–2018 period according to the National Cancer Institute Surveillance, Epidemiology and End Results program). It is characterized by abnormal B-cell clonal expansion and originates from the germinal center (GC). Immunotherapy has drastically improved FL outcome. Specifically, the use of anti-CD20 monoclonal antibodies (such as rituximab) has increased the patients’ overall survival to 90%. However, many patients experience relapses and develop treatment resistance, or transformation to more aggressive lymphoma types (usually diffuse large B cell lymphoma (DLBCL) in 30% of patients) [1, 2]. Hence, there is an urgent need of new clinical options for the long-term cure of FL. Approximately 90% of patients with FL harbor the t(14;18)(q32:q21) chromosomal translocation in which the immunoglobulin heavy chain (*IGH*) enhancer region at 14q32 and the B-cell lymphoma 2 (*BCL2*) gene at 18q21 are juxtaposed. This translocation is characterized by a transposition of the *BCL2* oncogene to the regulatory region of the *IGH* gene, thus leading to *BCL2* ectopic expression and constitutive activation of the anti-apoptotic program. In patients with FL, this translocation can be found also in bone marrow CD34^+^ CD19^+^ B-cell precursors [3]. However, the detection of *BCL2* genetic aberrations at low frequency (40%) also in healthy individuals is one of the many evidences supporting the hypothesis that this translocation is insufficient for FL development and that other genetic and epigenetic alterations (such as *BCL6*) are required [4–6].

Epigenetic processes are implicated in the regulation of gene transcription and translation. They include DNA methylation and histone modifications through the transfer of acetyl or methyl groups. Deregulation of such processes (e.g. aberrant DNA hypermethylation) has been recognized as a central feature of hematologic malignancies [7, 8], particularly FL where epigenome alterations have been observed in almost all patients [9, 10]. This could be explained by the multistep GC reaction process in which gene expression is finely regulated by post-translational histone modifications and/or chromatin remodeling. In FL, mutations in epigenetic-related genes concern mainly histone methyltransferases (*KMT2D* and *EZH2)* and histone acetyltransferases (*CREBBP* and *EP300*), and they are mostly inactivating mutations, except in *EZH2*. They play a crucial role in gene expression regulation by modifying lysine 4 on histone 3 (H3K4) (*KMT2D*) and lysine 27 on histone 3 (H3K27) (*EZH2*, *CREBBP* and *EP300*) [9, 11]. Moreover, these epigenome abnormalities are common in adult FL, but not in the rare pediatric nodal FL that has a more favorable prognosis [12]. The impact of these alterations varies in function of the B-cell development stage. At early stages, these mutations are sufficient to initiate lymphomagenesis, while at later stages, additional epigenetic gene mutations might be required to support malignant transformation [13].

At the cellular level, the multiple relapsing/remitting cycles that characterize FL are partly due to the presence of a common precursor cell (CPC) population enriched in these early epigenetic driver and recurrent mutations, particularly in histone modifiers. This “founder” population acts as a FL niche that sustains the tumor microenvironment (TME) reprogramming through molecular (genetic and epigenetic) mechanisms to promote anti-tumor immune evasion and transformation [10].

In studies on FL, genetic/epigenetic alterations and TME are often characterized separately; conversely, in this review we wanted to discuss the impact of the FL epigenome on TME functional plasticity to better understand this indolent cancer.

## The FL epigenome influences its microenvironment

Gene regulation by epigenetic mechanisms involves the addition or removal of chemical groups on DNA or histones, catalyzed by enzymes called ‘writers’ and ‘erasers’. Moreover, ‘readers’ are a distinct group of proteins that are recruited to specific regions of the genome through recognition of the epigenetic marks deposited or erased by the ‘writers’ and ‘erasers’. Then, readers can modify/adjust the chromatin state and regulate the expression of target genes. DNA methylation and histone modifications are the most common epigenetic modifications. The mechanisms through which epigenetic regulators regulate gene expression have been described elsewhere [14, 15], and will not be discussed in this review. Here, we will focus on the mutations of the histone acetyltransferase *CREBBP* and the methyltransferases *KMT2D* and *EZH2* that play a key role in the FL microenvironment, and have detrimental consequences on the anti-tumor immune response.

FL is a cancer addicted to epigenetic gene mutations that are detected in almost all patients (80%), and concern particularly the histone methyltransferases *KMT2D* (90% of patients) and *EZH2* (25%) and the histone acetyltransferases *CREBBP* (30–60%) and *EP300* (9%). They are frequently combined with other mutations, for instance in linker and core histone genes and chromatin remodeling complexes [16]. All these mutations lead to a modification of the epigenetic landscape in FL that supports tumor progression.

A specific feature of FL is its strong dependency on its microenvironment. This is explained by various mechanisms [17–19], but is essentially correlated to the epigenetic changes that occur in the early stage of tumorigenesis. Indeed, the epigenetic dysregulation detected in FL can affect the expression of genes that regulate quantitatively and qualitatively the TME cell composition, thus influencing antigen presentation mechanisms, immune signaling pathways, and cell recruitment to escape immune surveillance and maintain an immunosuppressive microenvironment [19–21]. Therefore, these epigenetic alterations substantially affect the patient outcomes by providing survival signals for cancer cells and by hindering the anti-tumor immune response (Table 1).

**Table 1:** Summary of what is known about the FL epigenome

1) FL is addicted to epigenetic mutations, thus representing a good model of epi-cancer.	
2) The most frequent epigenetic mutations in FL concerns genes that regulate the deposition of histone marks.	
3) Most epigenetic mutations occur early in FL lymphomagenesis.	
4) Mutations in the epigenetic regulators *KMT2D*, *CREBBP* and *EZH2* affect the FL microenvironment.	
5) Combination therapies targeting the FL epigenome and its immunological synapse represent promising future approaches for FL management.	

### CREBBP mutations affect antigen presentation

**Fig. 1:**
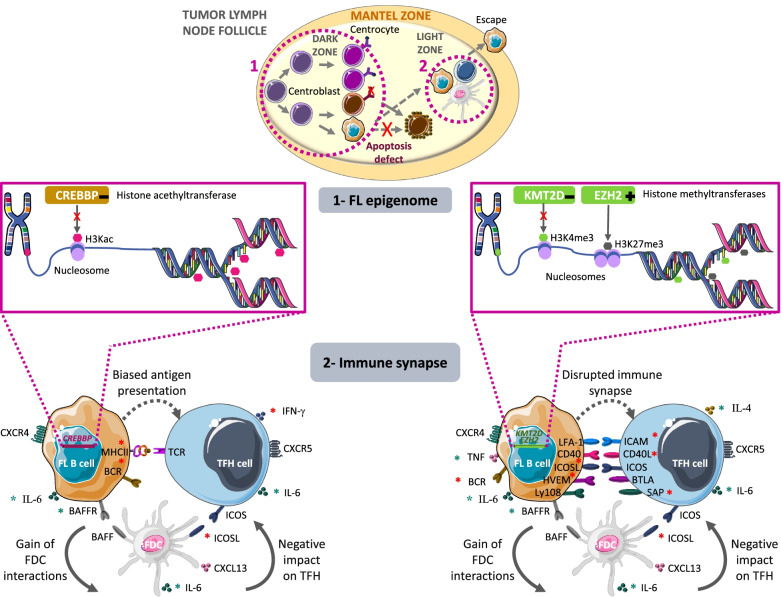
Epigenetic dysregulation in FL modifies its microenvironment. Somatic mutations occur during the early steps of FL lymphomagenesis (dashed circle, 1). Accumulation of genetic and epigenetic mutations promotes tumor progression. All these alterations accumulate during the different phases of the GC reaction, contributing to the dysfunctional B-T cell crosstalk (dashed circle, 2) that favors tumor growth, escape, and dissemination. 1- Schematic summary of the epigenetic regulators frequently mutated in FL: CREBBP, KMT2D and EZH2. These mutations occur concurrently in most FL, but it is not known whether they act alone or cooperatively in driving B cell malignancy and in shaping the FL epigenome. H3Kac, histone H3 lysine acetylation; H3K4me3, histone H3 lysine 4 trimethylation; H3K27me3, histone H3 lysine 27 trimethylation. Loss-of-function: -, gain-of-function: + . 2- Consequences of epigenetic gene mutations on the immunological synapse. Impact of *CREBBP*, *KMT2D* and *EZH2* gene mutations in FL B cells on the crosstalk of tumor cells, T follicular helper (TFH) cells, and follicular dendritic cells (FDC). Alterations of this cross-talk lead to inhibition of TFH immune synapse formation and antigen presentation, and increased FDC interactions. Specifically, *CREBBP* gene mutations affect antigen presentation, and *KMT2D* and *EZH2* gene mutations lead to a disrupted immune synapse. Red stars: decreased expression. Green stars: increased expression. Dashed arrows: abnormal pathway

In FL, the unbalance toward abnormal repression of gene transcription might be explained by the loss of histone 3 lysine 27 acetylation (H3K27ac; an active transcription mark) catalyzed by *CREBBP*. *CREBBP*, one of the most frequently mutated gene in FL (∼65%), encodes a lysine acetyltransferase that activates gene expression through acetylation of histone H3 lysine 18 (H3K18Ac), H3K27Ac, and other residues. Most *CREBBP* mutations lead to single–amino acid changes within the catalytic acetyltransferase domain that reduce its acetyltransferase activity [22]. FL sequencing identified *CREBBP* mutations as early oncogenic events, likely in the CPC founder population [23], thus partly explaining their association with poor outcome [24]. Interestingly, in FL, *CREBBP* mutations are correlated with reduced tumor expression of genes involved in the MHC class II antigen presentation pathway, and also with decreased B-cell receptor (BCR) and interferon signaling, accompanied by reduced proliferation of tumor-infiltrating T cells [23, 25]. Overall, as the lack of recognition and presentation of tumor antigens by immune cells contributes to tumor invasion and progression [25], these findings suggest that *CREBBP* mutations directly promote lymphomagenesis by controlling the antigen presentation and interferon signaling pathways (Fig. 1). Interestingly, MHC pathway deregulation is observed in the early stage of FL, and also in transformed FL and DLBCL. FL progression to a more aggressive form is associated with increased somatic hypermutation activity, leading to accumulation of mutations on several downstream targets [26, 27]. All these studies indicate that MHC deregulation is an important mechanism in FL pathogenesis and that it occurs at every FL stage.

### EZH2 and KMT2D mutations disrupt the immune synapse

*EZH2* mutations were the first epigenetic alterations to be reported in FL and to be investigated using murine models that showed their crucial role in early FL [28]. In FL, the unbalance toward abnormal repression of gene transcription is characterized by an increase in the repressive trimethylation of lysine 27 on histone 3 (H3K27me3) mark catalyzed by *EZH2*, the best characterized writer in lymphoma. *EZH2* is part of the polycomb repressor 2 complex, and is normally strongly expressed in GC B cells. Indeed, *EZH2* is one of the main regulators of the GC phenotype by repressing the expression of regulators of B-cell differentiation (such as *PRDM1* and *IRF4*) through H3K27me3 deposition [28]. *EZH2* mutations are the third most important recurrent gene alterations in FL, leading to increased H3K27me3 levels [29].

Recently, Beguelin et al. showed that in *Ezh2* conditional knock-in mice harboring the EZH2^Y641F^ mutation in the catalytic domain that increases H3K27me3 levels, GC B cells accumulate in the light zone because of the lack of the MYC-associated recycling signature, thus impairing cell re-entry in the dark zone and promoting terminal differentiation. An important effect of these *Ezh2* mutations is the prevention of the induction of genes (e.g. *Cd69*, *Icosl*, *Icam1, Icam2*, *Slam*, *Ly108,* and *Basp1)* that are normally upregulated in GC centrocytes. This is associated with a preferential spreading of the H3K27me3 mark from gene promoters to neighboring chromatin (due to their shared localization within topologically associating domains) [9]. As these genes are usually implicated in the formation of stable immune synapses with T-follicular helper (TFH) cells, this impairment results in the loss of the crosstalk with these cells and in the reduction of the CD40/CD40L response. Importantly, GC B cells harboring *Ezh2* mutations maintain GCs in the absence of CD40 signaling and TFH interaction thanks to their increased crosstalk with follicular dendritic cells (FDCs). This is possible through aberrant overexpression of genes involved in the interaction with FDCs, such as *Tnfrsf13c* (BAFF receptor), *Ltb* (FDC precursor) [9, 30–32]. In line with these observations, human FL with *EZH2* mutations have an atypical denser FDC network within follicles. Particularly, *EZH2* mutant GC B cells expand GCs until hyperplasia by enhancing centrocyte survival and by evading TFH-directed clonal selection and affinity maturation [9]. These studies suggest that *EZH2* mutations lead to aberrant repression of genes normally expressed during the GC reaction, thus preventing the normal clonal selection of GC B-cells, and allowing their survival and proliferation within the LZ by depending only on survival signals from FDCs. This reflects an epigenetic reprogramming that modifies the GC B cell interactions with TFH cells and FDCs, thus promoting malignant transformation and the establishment of the specific features of the FL immunological synapse and niche (Fig. 1). As GC B cells are very T-cell dependent, all these results might help to understand FL resistance to T-cell-based therapies.

*KMT2D* encodes a SET domain-containing lysine methyltransferase and catalyzes almost all mono-methylation of lysine 4 on histone 3 (H3K4me1). *KMT2D* is the most recurrently mutated gene in FL, resulting in its loss of function, thus promoting lymphomagenesis [33].

Transcriptomic analysis of a mouse model in which *Kmt2d* was ablated showed an abnormal enrichment in genes encoding immune-related proteins, such as CD40, JAK-STAT, Toll-like receptor (TLR), IL-6, TNF, and BCR signaling molecules. Among the *KMT2D* target genes, there is also the *TNFRSF14* (*HVEM*) tumor suppressor that is frequently mutated in FL (40% of patients) [13]. These mutations lead to the loss of the signaling cascade induced by HVEM interaction with its inhibitory receptor BTLA. Disruption of the HVEM/BTLA crosstalk causes hyperactivity of BCR signaling and consequently infiltration of BTLA^+^ TFH cells that produce high amounts of TNF-α, IL-4, and lymphotoxin a1b2 (crucial regulators of stroma differentiation and maintenance), reflecting an enrichment in stromal TME [34].

Interestingly, Haebe et al. performed single-cell analysis of FL samples from two sites/patient and found that in most patients, the two B-cell sub-clones grow separately with phenotypic divergences that correlate with TFH cell infiltration through the CD40-CD40L crosstalk, whereas the overall TME profile remains similar at both sites [35]. This discrepancy might be explained by the loss of the GC program flexibility and the ability to de-synchronize and adopt different functional circuits, independently of the original clone [36]. These studies suggest the existence of founder neoplastic clones with intrinsic characteristics to modulate the TME in favor of disease progression. Additionally, it has been reported that some gene alterations (e.g. cathepsin S, beta2 microglobulin, *CIITA* and *RRAGC*) [5, 27, 37–39] also support the establishment of a biased immune synapse in FL, independently of epigenome modifications. However, this is outside the scope of this review and will not be further discussed.

## Epigenetic modifying agents for FL treatment?

Due to FL indolence and slow progression, this malignancy is often diagnosed at an advanced stage with high tumor burden. The classical chimeric anti-CD20 monoclonal antibody rituximab is an effective immunotherapy, as single agent or in combination with first-line chemotherapy, to achieve long-term remission. Progression-free survival is variable because of the non-responsiveness of some patients [40].

Various agents are currently under investigation to restore the anti-FL response and to reduce/inhibit its supportive TME in order to improve the anti-tumor immunity by reprogramming immune cells [41, 42] (NCT02953509). Here, we discuss strategies to target epigenetic alterations that might restore the immunological synapse in FL.

Because of the high rate of epigenetic dysregulation in FL, epigenetic regulators represent targets of choice. Ongoing clinical trials focus on CREBBP, EZH2, and HVEM with promising responses in refractory/relapsed FL. Although the most common loss-of-function *KMT2D* and *CREBBP* mutations appear early in FL pathogenesis, they are hard to target. However, as CREBBP and p300*,* its closely related activator, regulate regulatory T cell (Treg) differentiation, the inhibition of CREBBP/p300 loss-of function mutations in FL might have some effects also on non-tumor cells within the TME [43].

As genes silenced upon *CREBBP* mutation are direct targets of the BCL6/HDAC3 tumor-repressor complex, the use of selective HDAC3 inhibitors (e.g. BRD3308) allows reversing CREBBP loss-of-function effects by restoring the immune response (T-cell anti-tumoral function optimization by inducing antigen presentation by MHC class II molecules, and interferon signaling) and inhibiting lymphoma growth [44].

The EZH2 inhibitor tazemetostat is the only epigenetic modifying agent approved by the US Food and Drug Administration. An early clinical trial in non-Hodgkin lymphoma **(**NCT01897571) showed its effectiveness, but with no alternative treatment options for refractory patients [45]. Although EZH2 inhibitors showed promising effects on the non-tumor microenvironment of other cancers [46–48], their effects on the non-tumor microenvironment of FL has not been investigated yet. Importantly, as *EZH2* mutations occur later in FL, they might not be the best therapeutic target for long-term cure. Moreover, EZH2 is physiologically involved in the GC reaction and in other important cellular mechanisms (e.g. T and natural killer cell development), agents that target mutated EZH2 in FL, rather than wild type EZH2, should be developed. In addition, the specific targeting of *EZH2* mutations is interesting to prevent the CPC population rebound correlated with FL relapses and transformation.

Importantly, mutations in these different epigenetic genes might interact and influence each other in FL. This would imply the concomitant use of different epigenetic modifying agents as a global epi-therapeutic strategy in FL.

Therapeutic combinations are among the best options to reduce the exceptional supporting role of the TME in FL and to improve the anti-tumor response. This could include the combination of epigenetic modifying agents with immunotherapies or other anti-cancer drugs. For instance, the response of HDAC3 inhibitors can be enhanced by combining them with anti-PDL1 immune checkpoint inhibitors to prevent interferon-induced adaptive immune suppression [49]. Recently, two CD19-directed CAR-T cell products (axicabtagene ciloleucel and lisocabtagene maraleucel) have been approved for B-cell lymphoma (DLBCL and FL at advanced stage), including in combination with an anti-CD20 monoclonal antibody and bendamustine, an alkylating agent [50–52].

All these preliminary results suggest that combination therapies synergistically improve FL outcomes, and that epigenetic modifying agents might be best used in association with other therapeutic agents (Table 1).

In conclusion, epigenetic modifying agents seems to be a very promising avenue for FL therapy; however, i) their complete response rate, as single agents, is still low; ii) their effects too broad; and iii) their toxicity is high due to their systemic delivery. This makes more difficult (as it is also the case with other therapeutic agents) to treat disease heterogeneity, non-responsiveness, and resistance to treatment [53]. High density lipoprotein (HDL)-based natural biomaterials could be used to concentrate their effects on the target and to minimize their toxicity, by associating the benefits of therapeutic targeting and controlled release of the active compound [54, 55].

## Concluding remarks

Currently, FL management is based on immunotherapies and chemo/radiotherapies, thus undervaluing the importance of epigenetic dysregulation and its strong impact on the TME. FL is addicted to epigenetic modifications and strongly relies on its microenvironment, suggesting a mutual impact and crucial role in FL pathogenesis. Understanding FL epigenetic landscape will require detailed genetic, epigenetic and transcriptomic approaches to determine how all these factors contribute independently to FL development. Moreover, the functional characterization of FL epigenome would help to dissect how the complex interplay of these alterations contributes to modulate the TME and leads to immunosuppression, thus to FL pathogenesis. This will also help to answer other outstanding questions (Table 2). Importantly, the presence of many epigenetic alterations in the CPC population increases the interest of epigenetic therapies for eradicating this founder clone in FL and preventing transformation. Due to the flexibility and reversibility of epigenetic modifications and the particular enrichment of epigenetic gene mutations in FL, they are perfect therapeutic targets and accurate biomarkers for early diagnosis. However, the complexity of the genetic/epigenetic alteration landscape within the same tumor, as a result of the concerted epigenetic regulator crosstalk, and the substantial impact of epigenetic drugs also on the non-malignant microenvironment may make it more difficult to predict the efficacy of epigenetic modifying agents in FL. Thus, for clinical translation, their effects must be restricted to the targeted tumor population and their off-target effects strongly reduced by using natural nanomaterials (such as HDL-based nanoparticles). Moreover, although epigenetic modifying agents represent an interesting therapeutic option for FL, their complete response rates, as single agents, have been low [56, 57]. Hence, combination therapies targeting the epigenetic dysregulations (upstream), the immunological synapse (downstream), and FL metabolic pathway would help to better attack all the pro-tumor mechanisms of FL. Lastly, the development of high throughput data analysis and whole sequencing will allow addressing important questions concerning the impact of FL epigenome on its complex informational immunological synapse. Ultimately, a personalized therapy based on each patient’s malignant epigenetic signature might be the best ways to improve FL outcome.

**Table 2:** Outstanding questions

1) How do mutations in the epigenetic genes *KMT2D*, *CREBBP* and *EZH2* (concomitantly or alone) orchestrate and promote the multi-scale (e.g. genetic, molecular, cellular) alterations that drive lymphomagenesis and CPC establishment in FL?	
2) What is the clonal evolution of CPC during FL course? How to properly define (in space and time) and target this population?	
3) How does FL develop from aberrant GC reactions driven by *EZH2* mutant-mediated reprogramming of the microenvironment?	
4) How do the epigenetic regulator-TME bidirectional crosstalk interfere with the therapeutic response in FL?	
5) Would it be possible to combine epigenetic modifiers that target tumor cells and non-tumor cells in the FL TME? If yes, what combination would be the best for long-term efficacy and complete cure?	

## Data Availability

Not applicable

## Abbreviations

FL: Follicular lymphoma
GC: Germinal center
TME: Tumor microenvironment
IGH: Immunoglobulin heavy chain
DLBCL: Diffuse large B cell lymphoma
BCL2: B-cell lymphoma 2
CPC: Common precursor cell
H3K18Ac and H3K27Ac: Histone 3 lysine 18 and 27 acetylation
BCR: B-cell receptor
H3K27me3: Histone 3 lysine 27 trimethylation
TFH: T-follicular helper
FDC: Follicular dendritic cells
H3K4me1: Lysine 4 on histone 3 monomethylation
TLR: Toll-like receptor
HDL: High density lipoproteins
